# An integrative collaborative care model for people with mental illness and physical comorbidities

**DOI:** 10.1186/s13033-020-00410-6

**Published:** 2020-11-11

**Authors:** C. Ee, J. Lake, J. Firth, F. Hargraves, M. de Manincor, T. Meade, W. Marx, J. Sarris

**Affiliations:** 1 NICM Health Research Institute, Western Sydney University, Penrith, NSW 2751 Australia; 2 School of Psychology and Translational Health Research Institute, Western Sydney University, Penrith, Australia; 3 IMPACT, Food & Mood Centre, Deakin University, Barwon Health, Geelong, Australia; 4 Professorial Unit, Department of Psychiatry, The University of Melbourne, Melbourne, Australia

**Keywords:** Integrative model, Collaborative care, Mental health, Comorbidity, Integrative medicine, Complementary medicine, Lifestyle medicine

## Abstract

**Background:**

Many individuals with mental health problems have comorbid physical conditions, or may present with substance/alcohol misuse or abuse issues. This results in complex treatment challenges that may not be adequately addressed by a model of care that is solely delivered by an individual clinician using a sole intervention. Mainstream pharmacotherapeutic treatment of mental health problems often have limited effectiveness in completely resolving symptoms, and may cause adverse side effects. Adjunctive treatment approaches, including nutraceuticals, lifestyle and behaviour change interventions, are widely used to assist with treatment of mental health problems. However, whilst these can be generally safer with fewer side effects, they have varying levels of evidentiary support. These circumstances warrant reframing the current treatment approach towards a more evidence-based integrative model which may better address the real-world challenges of psychiatric disorders and comorbid physical conditions. In essence, this means developing an integrative model of care which embodies an evidence-informed, personalized stepwise approach using both conventional pharmacological treatments alongside novel adjunctive treatments (where applicable) via the application of a collaborative care approach.

**Discussion:**

In order to inform this position, a brief review of findings on common patterns of comorbidity in mental illness is presented, followed by identification of limitations of conventional treatments, and potential applications of integrative medicine interventions. Advantages and challenges of integrative mental health care, collaborative models of care, review of research highlights of select integrative approaches, and comment on potential cost advantages are then discussed.

**Summary:**

We propose that a collaborative care model incorporating evidence-based integrative medicine interventions may more adequately address mental health problems with comorbid medical conditions. Robust research is now required of such a model, potentially within an integrative clinical practice.

## The current challenge

It is estimated that 300 million people globally are affected by depression, in addition to 60 million by bipolar affective disorder, and 23 million by schizophrenia [1]. Of concern, treatment for mental health problems is inadequate, with between 35 and 50% of people with mental disorders in high-income countries not receiving mental health treatment [2]. In low- and middle-income countries this figure is as high as 85% [3]. Further, delays in receiving care are common. On average, it takes almost 10 years to obtain treatment after symptoms of depressed mood begin, and more than two-thirds of depressed individuals never receive adequate care [4]. Between 10 and 20 million people with depression attempt suicide every year, and approximately one million will complete suicide [4]. Mental disorders such as depression, anxiety and schizophrenia are the largest cause of disability and economic burden across many countries [4].

Large population surveys of different world regions consistently show high prevalence of mental illness with other comorbid conditions [5] where they can be a precursor or a consequence of conditions such as major non-communicable conditions such as cardiovascular disorders, diabetes, respiratory conditions and cancer [4]. Actual rates of comorbidity in world populations are difficult to estimate and reported rates reflect disparate criteria used in different countries to classify symptoms into disorders [6].

Urgent unmet treatment needs in people with depression led the World Health Organization in 2016 to declare depression the leading cause of disability worldwide [7]. The need for treatment approaches that encompass both mental and physical domains to improve overall wellbeing and health of those experiencing depression has never been more evident. Achieving this requires adopting a more comprehensive evidence-based treatment model to address mental and physical health in a more integrated manner. This paper provides such a framework, firstly discussing the general background on medical comorbidity with mental illness, the current limitations of conventional mental healthcare, before outlining the potential benefits of integrative collaborative care, and providing a proposed model with clinical considerations for real-world application.

## Mental health disorders and physical comorbidity

Mental illnesses are frequently comorbid with a range of physical medical disorders in all age groups, and significantly impact the clinical course and response to treatment [8, 9]. In severe mental illness (SMI), which includes schizophrenia and other psychotic disorders and bipolar affective disorder, frequent physical co-morbidity results in a higher hospital admission rate for physical disorders than the general population, and a substantially lower life expectancy [10]. On average, people with SMI die twenty-five years earlier than the general population, with the primary cause of death being pathological illness rather than suicide [11]. In general, mental illnesses are associated with an increased risk of cardiometabolic conditions such as obesity, diabetes and cardiovascular disease at a rate 1.4-2.0 times higher than in the general population [9].

The reasons for co-morbidity are complex and intertwined, and include social determinants of health, lifestyle factors, sleep disturbance, and medication side-effects [9, 10]. Genetic studies also suggest that people with schizophrenia are more likely to develop metabolic syndrome, even if antipsychotic-naïve, although the evidence is inconsistent [11]. Likewise, physical comorbidities with depression are understood to be due to genetic and epigenetic factors [12]. It is well-established that antipsychotics have an orexigenic effect and can increase weight and promote dyslipidemia [13] as well as have a range of toxicities on haematological, neurological, and gastrointestinal systems [11]. Further, the rates of undertreatment of cardiovascular risk factors in people with schizophrenia are high [14]. Furthermore, many of the pathophysiological pathways implicated in mental disorders are also common in chronic diseases. These include heightened inflammatory [15] and oxidative [16] stress responses, mitochondrial dysfunction, and gut microbiota [17] dysbiosis. [15, 17, 18] and the association between depression and physical comorbidities such as cancer ,is likely due to shared aberrations in these pathways [19]. Older adults appear to be at greater risk of physical and mental multimorbidity. Last, poor mental health often results in lower capacity to implement lifestyle interventions [20], lower patient activation and engagement [21], social isolation (often due to stigma) [22] and low social capital [23], which increase the risk of chronic non-communicable disorders. Table 1 below describes some of the common comorbidities between mental health and physical conditions.

**Table 1:** Mental health conditions and physical co-morbidities

Co-morbid condition	
Cancer	One third of people with cancer meet diagnostic criteria for at least one psychiatric disorder, including major depressive disorder, anxiety disorders, adjustment disorders, sleep disorders and delirium [24]; prevalence is higher in people with advanced cancer Comorbid mental disorders reduce quality of life, interfere with treatment adherence and, in the case of depression, may affect the rate of cancer progression [25] However, people with schizophrenia are at lower risk of having cancer (OR 0.76) [26] People with cancer who have pre-existing depression have higher all-cause, cancer-related and non-cancer related mortality [27]
Chronic obstructive pulmonary disease	Individuals with chronic obstructive pulmonary disease (COPD) are at increased risk of depressed mood and anxiety compared to the general population [28]
Cardiovascular disease	Depressed mood and cardiovascular diseases frequently occur together with an estimated 20 to 45% of individuals with heart disease and depression and those who have had a heart attack 3 times more likely to be depressed compared to the general population [29] Relationship between heart disease and depressed mood is multifactorial. Risk factors include dysfunction of the hypothalamic-pituitary-adrenal axis, increased pro-inflammatory activity, reduced omega-3 fatty acids, reduced heart rate variability, smoking, physical inactivity and low self-esteem [29] Hypertension is one of the most common co-morbid conditions in people with schizophrenia [26]. People with schizophrenia have between a 1.2–3.6-fold increased risk of coronary artery disease, and up to 3-fold increase in sudden cardiac death than the general population [11]
Diabetes	Depression is associated with a 60% increase in diabetes and diabetes with 15% increase in depression [4] People with schizophrenia have an increased risk of diabetes (OR 2.23) [26] People with diabetes who report severe symptoms of depressed mood tend to be less compliant with treatment [30], and are at higher risk of coronary heart disease [31] Chronic hypoglycaemia caused by excess insulin secretion is often associated with intense anxiety and panic attacks [32]
Arthritis	People with depression have a 34% higher prevalence of arthritis than people without depression [33]
Alzheimer’s disease	Depression is a risk factor for developing Alzheimer’s disease (AD) and dementia symptoms [34], and treatment of depression in individuals with AD can improve cognitive function and quality of life [35]

## Limitations of conventional mental health care

Whilst there is substantial research to show that pharmacologic treatments can reduce the symptoms of major depressive disorder, bipolar disorder, and other psychiatric disorders, the evidence for complete and sustained remission from these treatments alone is limited [36–43]. Further, as many as one half of individuals being treated for psychiatric disorders fail to respond or respond only partially to psychotropic medications and are often labelled as “treatment-resistant” or “non-responders” [44–46]. Poor treatment outcomes due to limited efficacy of antidepressants, mood stabilizers, antipsychotics, and other psychotropic medications, alongside the socioeconomic, physical and environmental factors discussed in the section above, can result in long-term impaired functioning, work absenteeism, and losses in productivity [47–51]. Other biological therapies include electro-convulsive therapy, which is reserved for severe and complex depression and carries substantial risks [52], and transcranial magnetic stimulation, which has a variable clinical response [53].

When pharmacological treatment is not effective, an addition of psychotherapy is a recommended next step in the NICE guidelines [52]. A recent Cochrane systematic review [54] based on six randomised control trials found significantly greater short term and long term reduction in depressive symptoms for those who in addition to anti-depressant received psychotherapy. Given that depression often results from an interaction between biological, psychological and societal factors, a pharmacological treatment may only influence an aspect of the presentation, leaving other factors potentially unresolved and ongoing. With comorbidity between depression and other health conditions being more common than not, it is recommended that a network approach can be used, whereby treatment is designed to change or manipulate a symptoms network through *symptoms intervention* (e.g. interventions to treat low mood); *external triggers intervention* (e.g. interpersonal conflict resolution); and *network intervention* (e.g. cognitive behavioural therapy to address the impact of low mood on daily functioning) [55]. Further, as there is a high level of comorbidity between depression and other conditions, transdiagnostic-based treatments are likely to be more effective [56].

Alongside this, many commonly prescribed psychotropic medications including antidepressants and antipsychotics, are associated with serious adverse effects, including weight gain, metabolic syndrome, increased risk of diabetes and coronary artery disease, neurologic disorders, and sudden cardiac death [57], thereby in some cases being responsible for actually causing iatrogenic physical comorbidity. Further, although existing clinical guidelines provide some direction with regard to physical comorbidities and mental health [58–61], this guidance is generally limited to modifying treatment according to physical disabilities [58], prescription of metformin and statins for cardiovascular risk and obesity, and recommending cardiometabolic monitoring, close liaison with primary care, and lifestyle interventions [59–61].

## Non-pharmacological approaches to managing comorbidity

**Fig. 1:**
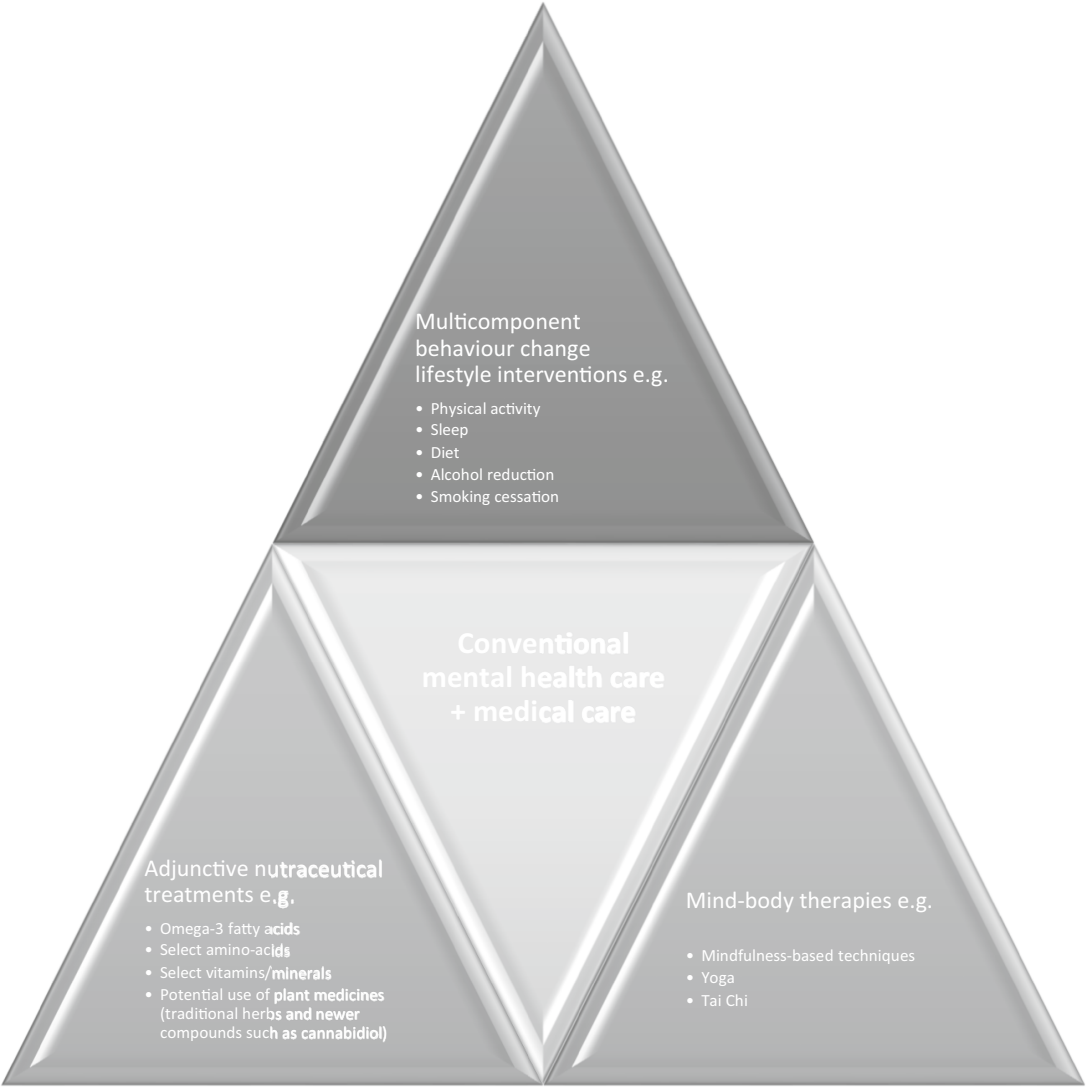
An integrative model of healthcare

There is a clear need for clinical approaches to healthcare that have the potential to improve both mental and physical health concurrently. In the context of the limitations of available treatment choices for mental health problems, increasing numbers of people are seeking concurrent treatment for their mental health from conventional medical and other practitioners utilizing approaches such as lifestyle medicine (e.g. diet, exercise, mindfulness practice), nutraceutical treatments, and complementary therapies [62, 63]. Whilst it is difficult to establish clear distinctions between these approaches, this may involve three key platforms, integrated with “conventional” mental health care, such as the appropriate prescription of psychotropic drugs and provision of psychological therapies e.g. cognitive behavioural therapy and medical care for co-morbidities. Further, ‘collaborative care’ approaches are emerging as a model of care that could improve outcomes in people with mental health disorders with physical co-morbidities [64]. Figure 1 below describes the platforms in our model.

1. *Multi-component behaviour change lifestyle interventions* combining exercise, diet, sleep, alcohol reduction, and smoking cessation interventions to improve lifestyle risk factors for both physical and mental illness [9, 65].
2. *Adjunctive nutraceutical treatments* such as omega-3 s, select amino-acids and vitamin compounds, along with potential use of plant medicines (including traditional herbs such as St. John’s wort and newer plant-based compounds such as cannabidiol/CBD) [66, 67].
3. *Mind-body therapies* such as yoga, tai chi, and mindfulness-based interventions [68].

## Potential role of lifestyle medicine and nutraceuticals

A number of lifestyle and integrative therapies have been evaluated for effectiveness as adjunctive therapies for mental health conditions such as depression and anxiety, and some show promise (see Table 2). These therapies have the potential to improve both mental and physical health, and therefore may improve health outcomes for people with mental and physical co-morbidities. In particular, there is a strong evidence base for the use of physical activity as a treatment for SMI, common mental disorders, ADHD and PTSD, as demonstrated within a recent meta-review of meta-analyses of RCTs; showing comparable efficacy across all of these conditions [69]. Whereas the evidence for dietary interventions is more nascent, there are now meta-analyses of RCTs showing small but statistically significant reductions in depressive symptoms from dietary interventions [70]. Interestingly however, the benefits so far seem to mostly be observed in predominantly female samples. Similarly, smoking cessation interventions can confer notable improvements for both physical and mental health in those with psychiatric disorders; as smoking is a leading cause of premature mortality in psychiatric populations, and reducing smoking also attenuates symptoms of depression and anxiety in those with mental illness [71]. The evidence for effectiveness of mind-body therapies such as yoga and mindfulness-based interventions for both mental and physical health is more preliminary in nature, however the literature is promising. There is the potential for mind-body therapies to impact positively not only on mental health symptoms such as depressive and anxiety symptoms, but also assist with adherence to behaviour change due to a greater acceptance of the discomforts of calorie restriction and increasing physical activity [72–75]. Most of these therapies have the advantages of being relatively low-risk with the exception of nutraceutical supplements which have the potential for more adverse effects and supplement-drug interactions. They can be integrated into the care of a person with mental and physical co-morbidities as adjunctive therapy.

**Table 2:** Biological, lifestyle, and mind-body medicines for mental health disorders and physical co-morbidities (Can be an appendices if required)

Therapy	Supporting evidence for effectiveness—mental health disorders	Supportive evidence for effectiveness—physical health
Exercise	A recent meta-review of exercise for the European Psychiatric Association’s guidelines confirmed significant symptomatic improvements for those with major depression or psychotic disorders, when used as an adjunctive treatment [118] Exercise may also provide effective first-line treatment for those with moderate depression [69] and may act as a preventative intervention; as physical activity significantly reduces the risk of developing depression or anxiety disorders [119, 120]	Exercise is associated with reduced risk of 26 difference types of cancer including breast, colorectal, esophageal, liver, and lung cancer [121]. Exercise training results in improvements in physical functioning, quality of life and cancer fatigue in several cancer survivor groups [122] Sedentary behavior is associated with greater risk for cardiovascular disease, cancer and type 2 diabetes [123] Exercise reduces the risk of cardiovascular disease and stroke in men and women by 20–30% and 10–20% respectively [124], of type 2 diabetes by 35% [125] and of all-cause and Alzheimer’s dementia by 10% and 13% respectively [126]
Mindfulness-based interventions (MBIs)	MBIs are more effective than treatment as usual, no treatment, non-specific active controls and specific active controls for common mental disorders, and similar in effectiveness to other active control conditions such as cognitive behavioural therapy [127, 128]. Effects in severe mental illness yet to be assessed. It should be noted that methodological quality of some studies in this field are poor, and overly enthusiastic conclusions may need to be tempered Yoga as an example: Moderate evidence for short-term effects for depression severity compared to usual care, limited evidence compared to relaxation, aerobic exercise [129] A 2013 meta analysis reported no evidence for any effect on positive or negative symptoms of schizophrenia, and moderate evidence for short-term effects on quality of life in high risk of bias studies [130]. A 2017 Cochrane Review reported Minimal differences between yoga and non-standard care (exercise), with the evidence too weak to draw any conclusions [131]	May help with adherence to lifestyle interventions. A number of meta-analyses suggest that mindfulness-based interventions may be beneficial in weight loss and in reducing obesity-related eating behaviours [72–75] Mindfulness-based interventions may be beneficial for people with diabetes by reducing depression, anxiety and distress symptoms, but their effect on physiological outcomes is mixed [132] Low quality evidence suggests mindfulness may have a small effect on improving chronic pain [133] Mindfulness-based interventions are beneficial for anxiety and depression symptoms in people with cancer [134] Randomised controlled trials have reported that mindfulness based interventions improve fatigue [135, 136] sleep quality [137] enhance psychosocial adjustment and quality of life [138] in women with breast cancer Promising evidence for cardiometabolic health (small changes in BP, lipids, BMI) [139] Reduces HbA1c by 0.39% compared to physical exercise in people with T2D [140] Recommended by the American Society of Clinical Oncology for anxiety, stress reduction, depression, mood disturbance, and improved quality of life in people with cancer [141] Improve health-related quality of life in individuals with substance abuse disorders [142], more effective than waitlist control for reducing consumption of illicit substances and alcohol although findings are limited by small sample sizes, inconsistent results [143]
Diet	Meta-analyses of RCTs confirm the benefits of dietary improvement for reducing symptoms of depression across the population [70]. In particular, three recent clinical trials have also reported the benefits of Mediterranean diet interventions on depression and depressive symptoms [144–146]	An umbrella review of observational studies and randomised controlled trials reported that greater adherence to the Mediterranean diet reduces risk for overall mortality, cardiovascular diseases, coronary heart disease, myocardial infarction, overall cancer incidence, neurodegenerative diseases and diabetes [147] Other examples include: An observational study suggests high adherence to the DASH, Mediterranean or a hybrid DASH-Mediterranean (MIND) diet may reduce risk of Alzheimer’s disease [148] Flavonol intake improves CV risk factors [149] Healthful plant-based diets reduce diabetes risk by 30% in a meta-analysis of observational studies [150] Vegetarian diets reduced HbA1c by -0.39% in a meta-analysis of six RCTs [151] in people with T2D Mediterranean diet was associated with better glycemic control and cardiovascular risk factors than control diets, including lower fat diets, in people with T2D [152] and shows strong and consistent beneficial effects on cardiovascular outcomes including clinically meaningful reductions in rates of coronary heart disease, ischemic stroke and total cardiovascular disease [153]
Nutraceuticals/phytoceuticals	Nutraceuticals: Recent meta-reviews [108] have found evidence supporting the use of: (i) omega-3 s as an adjunctive treatment for major depression or ADHD, (ii) bioactive forms of folic acid (i.e. l-methylfolate) in the treatment of psychotic disorders and major depression, (iii) N-acetyl cysteine for managing peripheral symptoms of severe mental illness, across a range of conditions Phytoceuticals: A systematic search identified 9 relevant meta-analyses of RCTs, with primary analyses including outcome data from 5,927 individuals. Supportive meta-analytic evidence was found for St John’s wort for major depressive disorder (MDD); curcumin and saffron for MDD or depression symptoms, and ginkgo for total and negative symptoms in schizophrenia. Kava was not effective in treating diagnosed anxiety disorders (Sarris 2020, in submission ENP)	CVD: Supplementing with tomato products (lycopene) has beneficial effects on BP, lipids and endothelial function [154]. Calcium may have a modest benefit for BP [155]. Moderate to low quality evidence supports the use of some nutrients for prevention of cardiovascular events (folic acid, B-vitamins) [156]. The use of omega-3 s for prevention of cardiovascular events has been controversial however the most recent meta-analysis of 127,477 participants demonstrates lower risk of cardiovascular events including AMI, CHD death, and total CVD death [157] with marine omega-3 supplementation. Coenzyme Q10 may be beneficial in heart failure [158] T2D: Zinc [159], curcumin [160], Coenzyme Q10 [161], probiotics [162, 163], and chromium [164] have been demonstrated to have beneficial effects on glycemic control and cardiovascular risk factors in meta-analyses in people with T2D

## Collaborative care

Collaborative care is a complex intervention that aims to foster close working relationships between members of a treating team, often involving the integration of primary and secondary health care, in order to provide high quality care to a patient and improve both mental and physical health outcomes. There is no single agreed on definition of collaborative care, however in essence collaborative care involves more than one health care professional providing care to the patient, and systematic efforts to improve communication and teamwork within the treating team. Apart from a multidisciplinary team, other components of collaborative care may include case management or coordination, patient education, provider education, systematic follow-up of the patient, use of guidelines and algorithms, psychological interventions, and shared decision-making with patients [10].

The integration of physical and mental healthcare providers in a collaborative model can improve the quality of care that is provided, and address both physical as well as mental health needs of the patient [76]. Outcomes studies support that collaborative care models are more effective than conventional care models for treatment of depressed mood, anxiety disorders, bipolar disorder, and schizophrenia [77–82] as well as treatment of individuals with comorbid physical conditions [10, 81, 83]. Collaborative care models also may reduce health care disparities in patients from different socioeconomic and ethnic backgrounds, thereby improving access to care [84–87].

People with depression or other serious health problems or comorbid presentations, often respond better and more rapidly when managed by a team of practitioners using collaborative care models. Collaborative care is more cost-effective than usual care in all categories measured, including medication costs and inpatient, outpatient, and mental health specialty care [88], as well as for the management of depressed patients with comorbid medical disorders [83, 89], severe anxiety disorders [90] and serious chronic mental illness [91–93]. Finally, both providers and patients report high levels of satisfaction with the management of depressed mood in collaborative care settings [78, 94]. The next step in advancing true collaborative care, is in the utilization of evidence-based integrative modalities within this treatment model.

## Integrative medicine

Integrative medicine is defined as a practice that “…reaffirms the importance of the relationship between practitioner and patient, focuses on the whole person, is informed by evidence, and makes use of all appropriate therapeutic and lifestyle approaches, healthcare professionals and disciplines to achieve optimal health and healing” [95] Approaches used in integrative mental health care may include prescription medication, psychotherapy, psychological treatments, nutraceutical and herbal supplements, mind body therapies, and positive lifestyle changes such as diet, exercise, and sleep hygiene.

Integrative mental health care may result in significant cost-savings. Findings from economic modelling research suggest that whilst a more integrative approach may initially cost more, down-stream savings can be achieved when integrative strategies yield positive long-term outcomes [96, 97]. Similarly, systematic reviews of economic modelling studies on comparative cost-effectiveness of conventional versus integrative treatments of many health conditions (including mental illness), suggest that integrative treatments are cost-effective, and in some cases, provide cost savings [42]. Individual lifestyle interventions have also demonstrated cost-effectiveness [98, 99]. Finally, higher up-front costs of integrative treatment approaches may be potentially offset by improved work productivity and increased future Quality Adjusted Life Years (QALYs) [96]. Further, cost-effectiveness of individual lifestyle interventions has also been demonstrated [98, 99].

Integrative medicine lends itself well to a collaborative care model, given the number of different modalities that could play a role in care of the patient, and the holistic approach that is involved. We propose a model for integrative collaborative care for people with mental and physical co-morbidities.

The term integrative medicine should be distinguished from *integrated care*, which has many definitions and associated models [100], but in this context may refer to vertical integration of primary and secondary care services.

## An integrative collaborative care model

In this section we develop practical clinical methods for integrative management of complex patients with high comorbidity in collaborative care settings. These methods are informed by qualitative research we conducted while designing the model of care for Australia’s first academic integrative healthcare centre [101, 102], and a health forum which was undertaken in Sydney in April 2019 to inform the direction of research arising from a White Paper on depression and co-morbidity commissioned by Western Sydney University [103]. To the best of our knowledge, no such model has been described in the literature. This model lends itself well to a primary care setting, as primary care health professionals (general practitioners/family physicians) are experts in provision of whole-person, comprehensive and person-centred care [104]. It is not uncommon for primary care practices to include a range of allied health and integrative medicine practitioners on-site, and this would serve as an ideal opportunity to build a collaborative care model. Alternatively, a community health setting, or secondary care setting, with appropriate integration with primary care and allied health, would also work well.

### Triage and assessing urgency

Initially, a clear written policy and procedure for triaging and managing psychiatric emergencies and psychological distress, including assessment and management of suicide risk, is required. This policy and procedure should be available to all members of the care team, as immediate risk from worsening mental health may be uncovered in consultations with allied health and complementary therapy practitioners. Similarly, clear written policies and procedures (along with necessary training and updating of skills) are required for triaging and managing medical emergencies such as acute myocardial infarction, stroke and diabetic ketoacidosis. This can include the identification of physical “red flags” in history, such as sudden onset chest pain on exertion, that should trigger urgent medical review.

### Creating a comprehensive care plan

In collaborative healthcare models, a comprehensive plan for individual patient care is developed by the healthcare team in partnership with the patient and their family and/or carers. This history may be taken in collaboration by several members of the healthcare team. There should be a designated team member who assumes responsibility for creating and disseminating the care plan. This could be the primary care practitioner, care coordinator, or mental health clinician (e.g. psychologist/psychiatrist).

### Clinical history

A collaborative care team can contribute to different aspects of history taking and assessment which should comprise a thorough documentation of all previous and current mental health, substance use and co-morbid physical problems. The process of onboarding and intake should be outlined clearly in policies and procedures, including which team member initially consults with the patient. Relative priorities are subsequently assigned to ongoing problems with respect to the amount of distress or impairment in functioning they cause, and prioritisation is done in collaboration with the patient, family, carers, and other health professionals. Some useful tools to facilitate this include the Measure Yourself Concerns and Wellbeing (MYCAW) questionnaire [105], What Matters Index, or the Bubble Diagram [106, 107]. Further formal assessment may be required to clarify physical and psychiatric diagnoses.

### Lifestyle habits

The key in providing excellent healthcare to people with mental health conditions and physical co-morbidities is the application of lifestyle medicine. A comprehensive lifestyle and social history should be obtained, including current and past physical activity levels, current ability to perform physical activity, preferences for physical activity options; intake of fruits, vegetables, processed foods, takeaway, alcohol, and soft drinks; availability of fresh food; sleep history; a social history including cultural background, employment, family and home support, social networks, ability to pay for treatment; and individual preferences and values regarding treatment. This information is crucial to obtain as ability to pay and personal preferences may impact on the treatment that is selected. Further, ability to perform physical activity needs to be factored into lifestyle recommendations, as this may be impacted upon by physical comorbidities e.g. poor mobility and balance from Parkinson’s disease. Last, availability of fresh food is limited in some geographical areas (often referred to as “food deserts”) therefore impacting on ability to make positive dietary changes. A comprehensive lifestyle history is facilitated by the involvement of team members with the relevant expertise, i.e. exercise physiology and dieticians.

### Informed decision making

Informed decision making involves identifying appropriate treatment modalities for consideration, with a discussion around the potential risks and benefits of available treatments (conventional psychiatric/psychosocial and medical, as well as adjunctive integrative therapies) in the process of obtaining informed consent. The process of informed decision making is enhanced by availability of guidelines, written clinical pathways and evidence syntheses. Patients and practitioners can only make this decision after considering the best available evidence on the proposed therapies, the potential risks (including supplement-drug interactions) and the patient’s individual circumstances and preferences. This discussion is especially important when considering complementary and integrative therapies, where evidence for efficacy may be emerging or scarce. The discussion should be documented in the medical record. The risks of treatment include the financial cost, which may be prohibitive in the case of lack of insurance and ability to pay. In these cases, group-based care may be helpful. Where nutraceuticals and herbal supplements are prescribed, potential adverse effects and supplement-drug interactions should be screened for prior to the patient commencing treatment.

### Treatment plan

The plan should detail the advice given, treatments that were agreed on, the duration and frequency of treatments, and details of follow-up and review. If nutraceutical and herbal supplements are recommended, these should be recorded in the prescription history, alongside pharmaceutical treatments. Where the recommended therapies are outside of the scope of conventional healthcare (e.g. yoga therapy), a brief description of the therapy and summary of evidence for effectiveness and safety can be a useful knowledge transfer exercise. A key aspect of collaborative care is the sharing of the treatment plan with all of the treating team.

### Efficacy and safety of nutraceuticals and herbal medicines

While detailed discussion of the evidence of individual nutraceuticals and phytoceuticals (herbal medicines) are outside the auspices of this review, readers are advised to consult these recent reviews of the area [66, 108, 109]. In summation, there are a range of natural products which have supportive RCT or meta-analytic level of efficacy, however quality and standardisation can be an issue, especially in regards to phytoceuticals. In respect to safety, a recent meta-review of nutraceuticals for mental health disorders (including polyunsaturated fatty acids (PUFAs), vitamins, minerals, antioxidants, amino acids and pre/probiotic supplements) reported that all nutrient supplements included in the review did not result in serious adverse effects or contraindications with psychiatric medications [108]. A number of important supplement-drug interactions however should be considered prior to prescription. A collaborative care approach, which includes a shared medical record and improved interprofessional communication, can facilitate early identification of potential adverse events and prevention of drug-supplement interactions. An important advantage of collaborative care settings over conventional care models is the opportunity for shared learning between conventionally trained physicians and other allied health practitioners about appropriate safe uses of a wide range of conventional and integrative modalities.

### Psychological therapies

Psychological therapies are a vital component of collaborative care in mental health, and are advocated to be provided by those with adequate high-level training. For example, the most evidence-based approach, cognitive behaviour therapy, is based on a premise that maladaptive cognitions are the cause of emotional distress and behavioural problems and that change can be mobilised through various cognitive (i.e. cognitive restructuring) and behavioural (i.e. pleasant activities) strategies that are effective across a range of mental health conditions and comorbid presentations [110].

### Assessment tools

A systematic approach to follow-up is often a key component in high quality collaborative care. The judicious use of anthropometric measures, physical activity tracking (using fitness trackers or accelerometers), and pathology, are highly recommended. In particular, regular cardiometabolic monitoring is essential. The use of validated Patient Reported Outcome and Experience Measures (PROMS and PREMS) are also recommended in order to objectively assess any change in clinical condition. These would depend on the individual mental health and physical conditions, but can additionally include generic measures of quality of life such as the EQ-5D [111] or PROMIS 29 [112]. Assessing the patient’s main clinical need can be undertaken through use of a tool such as the MYCAW [105]. Other measures that can be helpful include measures of patient activation (the Patient Activation Measure) [113] and chronic disease self-efficacy (the Self-Efficacy for Managing Chronic Disease Scale) [114].

Additionally, at all clinical encounters, adverse events should be enquired about and documented. It may also be necessary to monitor for and manage metabolic and other sequelae from pharmaceutical treatments. A care coordinator can play an important role in organising regular review based on clinical pathways and algorithms, and importantly in following up patients who do not attend scheduled follow-up.

### Patient education

In many collaborative care models, patient education is a key component. This can be delivered in several formats: written information, via mobile applications, or video (online or telehealth formats) can enhance the delivery of collaborative care. Written information on the benefits and risks of pharmaceuticals, nutraceuticals and phytoceuticals and on the importance of adhering to treatment and of regular monitoring of physical health are also recommended. Shared Medical Appointments or Group Medical Visits are emerging as a novel way to deliver healthcare [115]. Education about lifestyle changes can be combined with the shared medical appointment, and the addition of a group integrative therapy such as group acupuncture, mindfulness meditation, yoga therapy or tai chi may also be of benefit.

## Facilitating team-based care

Collaborative care improves on conventional healthcare by using a multidisciplinary approach and improving communication between health care professionals caring for a patient. The healthcare team may include a practice nurse or mental health nurse, primary care practitioner/general practitioner, psychiatrist, psychologists, other medical specialists e.g. neurologist/endocrinologist/cardiologist, allied health practitioners e.g. dieticians, exercise physiologists, physiotherapists, drug and alcohol services. Integrative collaborative care may also include complementary therapy practitioners on the healthcare team, where appropriate, such as yoga therapists or those specialised in natural product prescription. It is important that scope of practice for each practitioner is carefully delineated, and that appropriate credentialing of practitioners is implemented. Particular care may need to be taken in the case of health professions that do not have mandatory regulatory standards.

- *Care coordination *is a common component in collaborative care, and involves a designated person ensuring that the needs of the patient are met, and access to relevant services are facilitated [116, 117]. This includes coordinating referrals that are required to provide holistic care. The role of care coordinator or case manager could potentially be held by a practice nurse, mental health nurse, or health advocate.
- *Enhancing interprofessional communication* is an essential component of collaborative care. Utilising a shared medical record wherever possible is highly recommended. Sharing of the medical record and treatment plan may be facilitated through a universal patient-controlled health record, for example in Australia using the MyHealthRecord. Communication with other team members can be facilitated through “warm handovers” and secure messaging. In some cases, case conferencing may be useful, or joint consultations with the patient. The authors also see the value in opportunistic (discrete and de-identified) “corridor consultations”, which are facilitated by co-location of healthcare providers. Regular multidisciplinary team meetings to discuss new or complex cases are also recommended.
- *Other interventions* that can enhance team-based care include “shadowing”, experiential sessions (e.g. trialling yoga therapy or exercise physiology personally), and in-service sessions with practitioners presenting information on their modality to the team.
- *Physical integration* of primary and secondary healthcare can be helpful, for example having an on-site psychiatrist consult in the same centre as the primary care practitioner and allied/complementary health practitioners once a week, including the opportunity to hold joint case-conferencing.

## Research considerations

Whilst there is growing evidence within each of the modalities of adjunctive treatment above, there is also great potential for future research, in order to better inform evidence-based mental healthcare for people with physical co-morbidities. Our recommendations for future research priorities are as follows:

1. Research that focusses on a range of service delivery elements are needed, including: improving access to mental health services (particularly in public health systems); improving mental health literacy (for providers/patients/carers); identifying barriers to high quality collaborative care for people with mental and physical co-morbidities; supporting health service providers; delivering a person-centred approach;
2. Research in this area should involve the community and patient/consumer-informed study designs;
3. Physical Activity (PA): whilst there is now robust evidence from meta-analyses of RCTs supporting the use of exercise interventions in the treatment of mental illness, there is now a need to assess if PA interventions can effectively prevent mental illness from arising in at risk population. Already, there is extensive evidence from population studies that greater levels of PA are associated with reduced mental health risk, however whether or not PA interventions can reduce the incidence of mental illness in the real-world settings has yet to be established.
4. Dietary interventions: Initial trials for dietary interventions in those with moderate depression have presented promising results, supported by RCTs showing reductions in depressive symptomology across the general population from dietary improvement. However, further replication (using more robust controls) and expansion on the research around dietary interventions as an adjunctive treatment for those with diagnosed psychiatric conditions is needed.
5. Nutraceuticals/Phytoceuticals: The emergent evidence for a range of natural products exist. A range of these should be further explored in future trials, particularly in young people in the early stages of mental illness. Alongside this, further implementation research is required to establish how supplements with existing strong evidence for efficacy (such as high-dose EPA for major depression) can be integrated into standard care.
6. Mind-Body Therapies: there is growing interest in mindfulness, yoga, and other mind-body activities for promoting mental health. Despite some preliminary evidence of beneficial effects compared to treatment-as-usual, the extent to which mind-body therapies exceed the general intervention effects (i.e. compared to relaxation control conditions, or light physical activity such as walking) has yet to be demonstrated. Furthermore, benefits in those with severe psychiatric conditions have not been widely assessed. Given the feasibility and acceptability of mind-body treatments, further research is urgently warranted to assess how the potential benefits can be maximized and implemented in clinical care. One particular format for this approach is via video ‘Telehealth’ delivery which may allow for greater patient accessibility and compliance.

## Summary

Leveraging collaborative care in primary care settings and providing individualized evidence-informed integrative interventions that address mental health needs has the potential to improve both psychiatric and physical health outcomes. Due to the challenges outlined, it is now urgent that a more integrated patient-centred approach occur within the field of mental health, with a particular focus also on physical health. Both are intimately connected and influence each other, thus an approach which considers both body and mind as one interrelated nexus. While research underpinning such an approach, and the potential down-stream psychosocial and economic benefits are nascent, as outlined above, there is genuine promise in this approach, and thereby continuing development of this model is advocated. The end result will likely be more cost-effective solutions to complex mental health problems and enhanced overall health of the population.

## Data Availability

Not applicable.
